# JESTR: Joint Embedding Space Technique for Ranking Candidate Molecules for the Annotation of Untargeted Metabolomics Data

**Published:** 2024-11-25

**Authors:** Apurva Kalia, Dilip Krishnan, Soha Hassoun

**Affiliations:** 1 Department of Computer Science, Tufts University, Medford, MA 02155, USA; 2 Google Research; 3 Department of Chemical and Biological Engineering, Tufts University, Medford, MA 02155, USA.

## Abstract

**Motivation:**

A major challenge in metabolomics is annotation: assigning molecular structures to mass spectral fragmentation patterns. Despite recent advances in molecule-to-spectra and in spectra-to-molecular fingerprint prediction (FP), annotation rates remain low.

**Results:**

We introduce in this paper a novel paradigm (JESTR) for annotation. Unlike prior approaches that *explicitly* construct molecular fingerprints or spectra, JESTR leverages the insight that molecules and their corresponding spectra are views of the same data and effectively embeds their representations in a joint space. Candidate structures are ranked based on cosine similarity between the embeddings of query spectrum and each candidate. We evaluate JESTR against mol-to-spec and spec-to-FP annotation tools on three datasets. On average, for rank@[1–5], JESTR outperforms other tools by 23.6% - 71.6%. We further demonstrate the strong value of regularization with candidate molecules during training, boosting rank@1 performance by 11.4% and enhancing the model’s ability to discern between target and candidate molecules. Through JESTR, we offer a novel promising avenue towards accurate annotation, therefore unlocking valuable insights into the metabolome.

**Availability:**

Code and dataset available at https://github.com/HassounLab/JESTR1/

## 1 Introduction

Analyzing biological samples using *untargeted metabolomics*, where masses of thousands of metabolites within a biological sample are detected, presents unprecedented opportunities to characterize the metabolome. Annotation, the process of assigning chemical structures to metabolomics measurements, however is riddled with uncertainty. Naïvely, one can presume that the measured mass could be used to determine a metabolite’s molecular structure. However, a particular molecular mass can map to possibly thousands of candidate molecular structures that share the same chemical formula (e.g., there are 44,374 known molecular structures associated with *C*_12_*H*_18_*N*_2_*O*_2_).

**Fig. 1.**
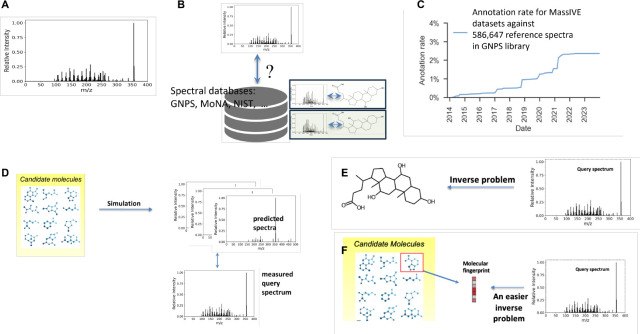
Current annotation workflows. A. Example spectrum measured using LC-MS or MS/MS, where x-axis represent the mass-to-charge ratio and the y-axis represent the relative intensity of each peak. B. Reference library search using spec-to-spec comparison. C. Current annotation rates using state-of-the-art library search are low despite growth in reference databases. D. Mol-to-spec predictive approach mimics the mass spectrometry fragmentation process. E. Spec-to-mol involves de novo molecular generation from spectra and forms an inverse problem. F. Spec-to-FP approach predicts a molecular fingerprint and identifies the candidate structure that most likely matches the predicted fingerprint.

With advances in instrumentation, it is now possible to not only measure the mass of ionized molecules, but to also measure masses of ionized molecular fragments. Combining liquid chromatography (LC) with mass spectrometry (MS) or combining two such analysis steps (tandem MS/MS) have now become dominant in metabolomics. The measured mass spectrum is a collection of peaks (Figure 1A). Each peak is represented by its mass-to-charge (m/z) ratio, where the charge is known and is often +1 or −1, and a relative intensity. Even for an experienced analytical chemist, assigning a chemical structure to LC-MS or MS/MS spectra is an unsolved problem as the spectrum provides a partial view on the measured molecule. Indeed, only ions that are formed by the loss or a gain of a charge can be detected by mass spectrometry.

Several techniques address the spectra annotation problem. The go-to technique is “spec-to-spec” comparison (Figure 1B) of the measured query spectra against spectra that are cataloged in spectral reference libraries (Kind et al., 2018). However, despite the growth in spectral libraries, e.g., GNPS (Wang et al., 2016), NIST (NIST, 2020), MoNA (MoNA, 2024), annotation rates remain extremely low due to the imitated coverage of spectral libraries in comparison to the space of all potential molecules. In addition, measured spectra vary tremendously under differing instrument settings, e.g., ionization energy, solvent type, and adduct formation (additional functional groups attached or removed from the ionized molecule). A molecule therefore may have many corresponding spectra, which further limits library coverage. A recent search of spectra within 15,327 datasets (Wang et al., 2016) deposited in the MassIVE (Mass Spectrometry Interactive Virtual Environment) database against 586,647 reference spectra cataloged in the GNPS reference library yielded a positive identification rate of 2.3% (Martin et al., 2024) (Figure 1C).

Two types of supervised predictive annotation techniques have emerged. “Mol-to-spec” techniques (Figure 1D) utilize combinatorial fragmentation approaches, e.g., MetFrag (Wolf et al., 2010; Ruttkies et al., 2016) and CFM-ID (Wang et al., 2021), MLPs (Wei et al., 2019), or GNNs (Zhu et al., 2020; Li et al., 2024; Young et al., 2021), to translate a molecular structure into a predicted spectrum. Candidate molecular structures are retrieved by either chemical formula, if available, or molecular mass from large molecular databases such as PubChem, or more biologically relevant, smaller, databases. The candidate with the most similar spectrum to the query spectrum is ranked highest and used as the annotation. In contrast, “spec-to-mol” techniques (Figure 1E) aim to generate *de novo* molecular candidates that potentially match the query spectrum, e.g., MSNovelist (Stravs et al., 2022), Spec2Mol (Litsa et al., 2023), MS2Mol (Butler et al., 2023). For example, MS2Mol uses sequence-to-sequence transformers to translate spectra into *de novo* molecular structures in the form of SMILES strings. Due to their current limited capabilities, *de novo* generation is presently of limited use in the metabolomics community. An alternative and earlier approach is “spec-to-FP” (Figure 1F), where a molecular fingerprint (FP) vector is predicted for the query spectrum, e.g., Sirius (Dührkop et al., 2019), MIST (Goldman et al., 2023). Here, the predicted fingerprint is compared against those of the candidate molecular structures, and the best match, via Tanimoto or cosine similarity, is declared the annotation result. Despite recent advances in all such techniques, annotation rates remain low as the *reconstruction* of spectrum, fingerprint, or molecular structure is a difficult task.

**Fig. 2.**
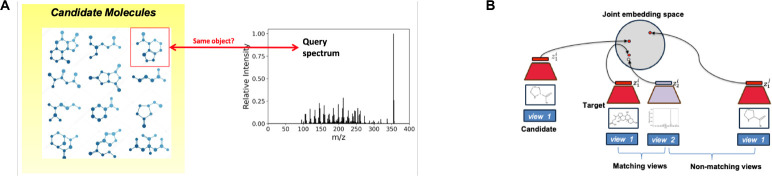
Novelty of the JESTR annotation approach. A. JESTR avoids the explicit generation of spectra, molecules, and fingerprints, and ranks the candidate molecules against the query spectrum based on their joint-space embeddings. B. JESTR learns to place representations of matching molecule-spectrum pairs close in the joint embedding space relative to non-matching pairs. Further, JESTR utilizes additional molecules beyond those in the training set to learn to distinguish target molecules in the training dataset from candidate molecules (those with similar molecular formulas).

We address in this paper the problem of assigning chemical structures from a candidate set to spectral data. The novelty of our approach lies in avoiding the explicit generation of molecular fingerprints and spectra (Figure 2A), and in considering a molecule and its spectra as views of the same object (Figure 2B). This valuable insight allows us to embed molecules and their matching spectra close in the molecule-spectrum joint embedding space. Our approach avoids the need for any kind of reconstruction to intermediate forms such as fingerprints or spectra and therefore removes any reconstruction loss that would invariably creep into the ranking pipeline with any reconstruction based approach. The ranking of candidate structures can be attained by comparing their embeddings against that of the query spectrum and selecting the candidate with the highest cosine similarity. The idea of learning joint embedding spaces from multiple views of the data dates back to the seminal work on Siamese Networks (Chopra et al., 2005). More recently, CLIP (Contrastive Language- Image Pre-training) was trained to create a shared embedding space for both images and text, enabling the model to match relevant images and captions without the need for direct labeling or supervised training on specific dataset (Radford et al., 2021). As we embed molecules and spectra in a joint embedding space, our method is termed Joint Embedding Space Technique for Ranking candidate molecules, JESTR. We use CMC, Contrastive Multiview Coding (Tian et al., 2020), to learn view-invariant information across different views of the data and produce embeddings in a joint embedding space. Recent work in contrastive learning (Chen et al., 2020; Tian et al., 2020; Khosla et al., 2020) has extended the Siamese network approach significantly. Broadly speaking, in all these approaches the key question is how to generate the paired views (either appearing naturally or generated via data augmentation); and how to ensure that paired views end up close together in the joint embedding space.

Another novelty of our approach lies in utilizing regularization on additional data consisting of millions of molecules with the same chemical formulas as those in the training dataset. While this form of data augmentation does not contribute directly to additional labeled training data (Tian and Zhang, 2022), the additional data is utilized to distinguish target molecules from their candidates. Here, regularization is used as a fine-tuning strategy towards the end of training. When combined with contrastive loss, regularization with additional data provides two key benefits: it improves model generalization by training on a larger, more diverse set of molecules, and it enhances representation learning for both molecule and spectra embeddings by using non-congruent pairs as additional data during training.

We conduct experiments and analysis to answer the following research questions. (Q1) Does JESTR’s implicit annotation method (with and without regularization) outperform prior explicit methods (mol-to-spec and spec-to-fp)? (Q2) Is learning the molecule-spectrum joint embedding space effective for distinguishing target molecules among their respective candidate sets? We compare JESTR against state-of-the-art mol-to-spec technique, ESP (Li et al., 2024), and spec-to-FP technique, MIST (Goldman et al., 2023). We conduct the evaluation using three datasets: the NPLIB1 dataset that was previously released with the CANOPUS tool (Dührkop et al., 2021), the well-curated, available-for-purchase NIST2020 dataset, and user-deposited data from MassBank of North America (MoNA) (MoNA, 2024). The contributions of this paper are:

- Novel implicit formulation of the annotation problem to avoid explicit prediction of spectra and fingerprints that has dominated the field since earliest attempts in solving the problem (Heinonen et al., 2012). Our formulation is grounded in the novel insight that molecules and spectra are views of the same object, similar to recent advances in linking text/image data.
- Demonstrating that contrastive learning is effective in creating a molecular-spectra joint embedding space, and that cosine similarity of the embeddings is sufficient for ranking candidate molecules. That is, there is no need for an explicit (learnable) downstream ranking task.
- Fine-tuning the implicit model via regularization using the candidate sets of the training molecules improves the rank @1 performance in the range of 6.04% to 37.11% when compared to a baseline that does not utilize regularization.
- Demonstrating that JESTR outperforms ESP and MIST on all ranking metrics and all datasets with the exception of rank@1 for the MoNA dataset. For rank@[1 through 5], JESTR outperforms ESP by 71.6% and MIST by 23.6% across three datasets. These remarkable improvements are achieved even though JESTR does not utilize the additional data in the form of chemical formulae labels for spectra peaks that is currently used by MIST.

## 2 Methods

**Fig. 3.**
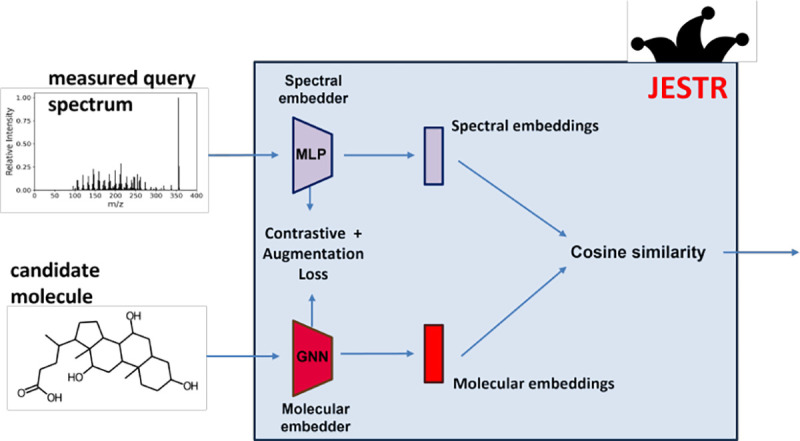
Overview of the JESTR model architecture. The model is trained to minimize the contrastive and regularization losses. The embeddings produced by the encoders are used to compute the cosine similarity in the joint embedding space between a molecule and a spectrum.

The JESTR model architecture (Figure 3) consists of a molecular encoder and a spectral encoder. They are trained to create embeddings in a molecule-spectrum joint embedding space. To place views of the same object close to each other in the embedding space, we use the CMC contrastive learning loss (Tian et al., 2020). To improve performance, we utilize regularization. At inference, when provided a candidate set for the query spectrum, the cosine similarity is computed between each candidate and the query spectrum. The candidates are then ranked based on their cosine similarities.

### 2.1 Encoders

The molecular encoder is implemented using a multi-layer Graph Neural Network (GNN) encoder. Molecular structures are encoded as graphs, where node features are include atom type, atomic mass, valence, if the atom is in a ring , formal charge, radical electrons, chirality, degree, number of hydrogens and aromaticity. Edge features are the bond type, whether the bond is part of a ring, conjugacity and one hot encoding of the stereo configuration of the bond. The encoder consists of Graph Convolutional Networks (GCNs) (Kipf and Welling, 2016) that aggregate information at each node. The GCNs are followed by a pooling layer and two fully connected layers to generate the final molecular embeddings, $z_{mol}$, for a given molecule graph c:

(1) $$z_{mol}=MLP_{\times 2}(MAXPOOL(GCN(c)))$$

To prepare the spectrum for its encoder, peak m/z values of the spectra are discretized into bins that are 1 Da wide. Peaks with m/z values larger than 1000 Da were dropped. The intensity are normalized to a max value of 999 - a common practice in normalizing spectral data (e.g., for the NIST datasets). For multiple peaks falling within the same bin, peak intensities within each bin are summed to generate the overall intensity value for that bin. A 1000-dimension binned vector therefore encodes the spectrum. A log10/3 transformation is applied to this binned vector to ensure that a few peaks and/or a long tail do not dominate the embedding vector. This 1000-dimension encoded vector was passed through a 3-layer MLP to obtain the final spectral embedding, $z_{spec}$:

(2) $$z_{s}=\frac{1}{3}{log}_{10}\left(\left\{\Sigma I_{i},\forall \;i,n<(mz{)}_{i}<(n+1),\;for\;n\;in\;0..999\right\}\right)$$

where $I_{i}$ is the intensity of the i-th peak and $mz_{i}$ is the m/z value of the i-th peak.


(3) $$z_{spec}=MLP_{\times 3}\left(z_{s}\right)$$

### 2.2 Contrastive learning of spectral and molecular views

We consider two views of each data item: a molecular and a spectral view. Each data item will have one molecular view but may have multiple spectral views as measurements may be collected under different mass spectrometry instrumentation conditions. Matching molecule-spectrum views arise from a molecule and its spectrum, while non-matching views arise between a molecule and any of its non-matching spectra. The objective of contrastive learning on multi-views (Chopra et al., 2005; Chen et al., 2020; Tian et al., 2020; Khosla et al., 2020) is to learn embeddings that separate samples from matching and non-matching distributions, and to ensure that paired views are close in the joint embedding space.

As in CMC (Tian et al., 2020), we use a discriminator function, h, to measure the closeness of spectral and molecular embeddings using their cosine similarity, modulated by a temperature parameter τ. Thus, given a embedding for spectrum n, and an embedding for molecule m, we can define h as:

(4) $$h(z_{spec}^{n},z_{mol}^{m})=exp(\frac{z_{spec}^{n}\cdot z_{mol}^{m}}{∥z_{spec}^{n}∥\cdot ∥z_{mol}^{m}∥}\cdot \frac{1}{\tau })$$

The hyper-parameter τ controls the importance of non-matching pairs in pushing the embeddings apart in the joint embedding space. To ensure that the discriminator assigns high values for matching pairs and low values for non-matching pairs, we define a contrastive loss, $L_{contrastive}$, over a batch of size k as:

(5) $$L_{contrastive}=\frac{1}{k}\sum_{n=1}^{k}\;\left[-E[log\frac{h_{\theta }\left(z_{spec}^{n},\;z_{mol}^{n}\right)}{\sum_{m=1}^{k}\;\;h_{\theta }\left(z_{spec}^{n},\;z_{mol}^{m}\right)}]\right]$$

This loss effectively ensures that the cosine similarity between each matching pair, $\left(z_{spec}^{n},z_{mol}^{n}\right)$, is highest among all possible pairings, $\left(z_{spec}^{n},z_{mol}^{m}\right)$, within the batch.

### 2.3 Regularization

As candidate molecules typically have the same molecular formula as the target molecule, we fetch such candidates from the PubChem database, and utilize regularization to train the model to better distinguish between target molecules and their candidates. Our regularization objective is therefore to push candidates away from the spectra, and hence from the corresponding target, in the joint embedding space. Training using regularization is implemented by introducing an additional loss to minimize the cosine similarity between the embeddings of each spectrum and the candidate molecules of the corresponding target. Candidate sets are sorted by their Tanimoto similarity to their respective target molecule. For each spectrum within a training batch, we chose a set of candidates given by the batch parameter, $k_{aug}$. Candidates selected for regularization in each batch are therefore the $k_{aug}$ most similar candidates, and are taken sequentially in each training epoch. Figure S1 in the Supplementary Information demonstrates the batching process for computing the regularization and contrastive losses. We then explored when and how to incorporate the regularization loss with our contrastive loss. Since our final ranking predictions are made using the molecular-spectral similarity in the joint space, the regularization attempts to push the most similar candidates away from the target molecule by minimizing a regularization loss function in addition to the contrastive loss. The regularization loss function minimizes the cosine similarity between the most similar candidates and the associated spectra - and hence the associated target molecules. The regularization loss, $L_{regularization}$, is defined as:

(6) $$L_{regularization}=\frac{1}{k}\sum_{n=1}^{k}\;\frac{1}{k_{aug}}\sum_{m=1}^{k_{aug}}\;\left(cosine\_sim\left(z_{spec}^{n},z_{cand}^{m}\right)\right)$$

The total training loss, $L_{total}$, is the sum of the two losses weighted by hyper-parameters α and β:

(7) $$L_{total}=\alpha \;*\;L_{contrastive}+\beta \;*\;L_{regularization}$$

We explored values for the α and β parameters, and we observed that utilizing regularization as a fine tuning strategy towards the end of the training provided the best performance. Regularization was turned on for the last 3% of the training epochs. The weight given to regularization loss was 10% to ensure that the matching pairs are not pushed too far apart during regularization. Therefore, α=1.0,β=0.0 for first 97% epochs and α=0.9,β=0.1 for last 3% of epochs. Implementation details and various hyperparameters are provided in Section S2 of Supplementary Information.

## 3 Results

### 3.1 Datasets

The NPLIB1 dataset was first utilized by the CANOPUS tool to predict compound classes, e.g., benzenoids, phenol ethers, and others, from spectra, thus providing partial annotation on spectra in cases when spec-to-spec comparisons in reference spectral databases yield unsatisfactory matches (Dührkop et al., 2021). This dataset was created by selecting spectra from the NIST2020 (NIST, 2020), GNPS (Wang et al., 2016) and MoNA (MoNA, 2024) databases. The selection ensured a desired distribution of compound classes. This dataset was recently renamed to NPLIB1 (Goldman et al., 2023) to distinguish it from the CANOPUS tool. We utilized the NPLIB1 data as assembled by MIST (Goldman et al., 2023). This dataset comprised 8,030 spectra measured under positive mode (positively charged, with an H adduct, [M+H]+) belonging to 7,131 unique target molecules. We utilize the same data split as proposed by MIST, where the split was structure-disjoint such that a molecule with the same InChiKeys did not appear both in the training and test sets. Therefore, 714 molecules and their 819 spectra were utilized for testing. Two additional datasets were utilized to explore training JESTR on larger datasets. The NIST2020 dataset is well-curated spectral database released by the National Institute of Standards and Technology. NIST2020 comprises a variety of molecules from human, bacteria, environmental, plant, and food samples. A variety of instruments and settings are used to measure spectra for each compound. The measurements are repeated and a consensus spectrum is created for each measurement. The NIST datasets are available under a commercial license, and we had access to the NIST2020 version of this dataset. The MassBank of North America (MoNA) is a collaborative database, with contributions by users. Both experimental and in-silico spectra are accepted. Here, we only retrieved the experimental dataset. Statistics for the three datasets is provided in Table 1. The number of unique molecules is largest in NIST2020, while the number of spectra per molecule is the lowest in NPLIB1. The splits for the NIST2020 and MoNA were created ensuring that no molecules overlapped between the training and test sets.

As typical in prior works (Dührkop et al., 2015; Huber et al., 2021; de Jonge et al., 2023), we select candidates for each target molecule in the training and test data by retrieving molecules from PubChem (Kim et al., 2019), by matching formulae of the target molecules. The average candidate sets for the target molecules range from 1,322 to 2,494 molecules, and for regularization, the average candidate sets for training molecules ranged from a 1,390 to 2,322 (Table 1).

### 3.2 Other annotation tools

To compare with other annotation models, we trained ESP (Li et al., 2024) and MIST (Goldman et al., 2023) on each of the datasets. The ESP model utilizes a GNN-based molecular encoder and an MLP on the molecular fingerprint. ESP is trained to learn a weighting between the molecular and fingerprint representations to predict the spectra. The best ESP performing model on rank@1 was the version that utilized the fingerprint and modeled peak co-dependencies, ESP MLP-PD. MIST first assigns chemical formulas to peaks within each spectrum using SIRIUS (Dührkop et al., 2019), and represents a spectrum as a set of chemical formulas. MIST trains a transformer model to learn peak embeddings and to predicts fingerprint. MIST also featurizes pairwise neutral losses and predicts substructure fragments as an auxiliary task. We ran both MIST and ESP on all three datasets, and confirmed the results with the respective teams.

### 3.3 JESTR vs explicit-construction models

Given a query spectrum, the primary task of JESTR is to identify the target molecule among a set of candidates. Candidate ranking was therefore selected as the performance metric. The rank@1, rank@5 and rank@20 indicates the percentage of target molecules that were correctly ranked within the top 1, 5 and 20 candidates, respectively.

**Fig. 4.**
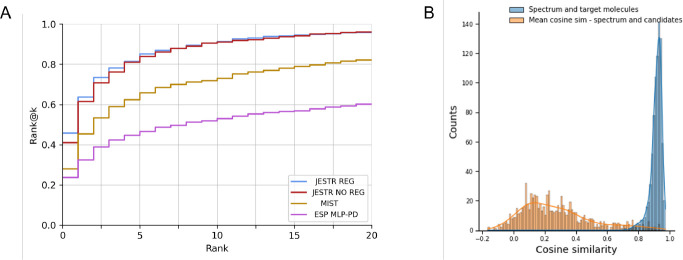
Results on NPLIB1. A. Rank@k results for JESTR , with and without regularization, ESP MLP-PD, and MIST. B. Distribution of cosine similarities of query spectra and target/candidate molecules with contrastive learning using JESTR.

JESTR is compared against ESP and MIST (Table 2A). For the NPLIB1 dataset, JESTR outperforms ESP and MIST on all reported ranks. For rank@1, JESTR outperforms ESP by 93.2%, and MIST by 64.1%. Further, JESTR achieves 95.8% rank@20, while the maximum performance of ESP and MIST is at 60.2% and 82.1%, respectively. We plot the detailed ranks for the NPLIB1 dataset (Figure 4A). At all ranks, JESTR provides superior performance to both ESP and MIST. For the NIST2020 dataset, JESTR outperforms all other models. Specifically, JESTR outperforms MIST at rank@1 by 83.8%. Detailed ranks for NIST2020 is provided in Figure S2A in the Supplementary Information. For the MoNA dataset, JESTR consistently outperforms ESP. MIST only outperforms JESTR at rank@1 by 17.6%, but not on rank@5, rank@20, or any other rank, as provided in Figure S3A in the Supplementary Information. Across all datasets, on avarage for ranks@[1–5], JESTR outperforms ESP by 71.6% and MIST by 23.6%.

Examining the overall performance of JESTR over the three datasets, we note that JESTR’s performance was worse on the MoNA dataset when compared to NPLIB1 and NIST2020. We suspect that JESTR’s performance on MoNA was low for two reasons. First, MoNA has the fewest number of molecules, thus providing the lowest molecular diversity among the three datasets. Second, MoNA data is uploaded by users and may not have undergone consistent curation efforts. The NIST2020 dataset is well curated; however, it has the highest ratio of spectra per molecule, an average of 13.25 spectra per molecule, versus 1.13 and 5.28 spectra per molecule for NPLIB1 and MoNA, respectively. As such, we suspect that JESTR finds it hard to place all the spectra embeddings closer to the molecule in the joint space for NIST2020 and MoNA. A combination of high molecule to spectra ratio, lower diversity of molecules and inconsistent spectra curation makes JESTR perform the lowest on MoNA. MIST, with additional information in the form of subformulae annotation on peaks, does a better job at distinguishing among various spectra of the same molecule for MoNA, thereby attaining a better rank@1 score on this dataset. However, MIST looses its advantage over JESTR starting with rank@2.

### 3.4 Joint-space embeddings distinguish target molecules from their candidates

The contrastive loss used in training JESTR ensures that the embeddings for matched spectrum-molecule pairs are placed close to each other in the joint embedding space, while non-matching spectrum-molecule pairs are placed further away. Figure 4B shows the distribution on spectrum-molecule cosine similarities for matching and non-matching pairs in the NPLIB1 test set. The corresponding distributions for NIST2020 and MoNA datasets are shown in Figure S2B and S3B respectively in the Supplementary Information. While any molecule other than the target can be considered a non-matching partner for the query spectrum, Figure 4B only considers candidate molecules (same chemical formula as the target) as the non-matching partner. It is clear that JESTR well discriminates between target and candidate molecules.

### 3.5 Ablation study - removing regularization

To assess the value of regularization, the model was retrained without . Regularizing the training loss with molecular candidates improves rank@1 by 11.4%, 6.0% and 37.1% on the NPLIB1, NIST2020 and MoNA datasets, respectively. Improvements using regularization is evident at almost all ranks and all datasets ((Table 2B). For rank@20 on NPLIB1, the results drop by 0.3% since the rank@20 result for NPLIB1 is high even without regularization, where even a small change in a few targets changes the result slightly.

**Fig. 5.**
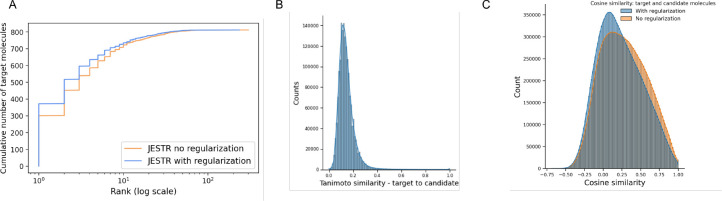
Regularization analysis for JESTR for NPLIB1. A. Regularization improves rank@k by significantly placing more targets at rank 1. B. Distribution on Tanimoto similarities on the ECFP fingerprints between target and candidates in the training set. C. Distribution on cosine similarities, with and without regularization, of the target and candidates within the test set.

To further demonstrate the value of regularization, we performed additional analysis on the NPLIB1 dataset. With regularization, the number of target molecules ranked @1 increases significantly, from 301 to 375, causing a ripple effect in improving other rank@k numbers (Figure 5A). Further, we examined the Tanimoto similarity between molecules in the training set and their candidates. We retrieved 15.86 million candidates from PubChem based on the chemical formulas of the target molecules in the training set. The majority of these candidates show low Tanimoto similarity with the target molecules (Figure 5B). Hence, our fine-tuning regularization strategy and sorting the candidates by their cosine similarity to the target effectively prioritizes regularization with the most similar candidates. We approximately utilized 7 million candidates for regularization during the last 3% of training epochs. Upon examining the cosine similarity distributions on the embeddings of candidate and target molecules (Figure 5C), we see that our regularization strategy reduces the average cosine similarity between targets and their candidates. Regularization is therefore effective, enabling the model to discriminate between a target molecule and its candidates. Similar analysis for the NIST2020 and MoNA datasets is shown in Figures S4 and S5 in the Supplementary Information.

## 4 Conclusion

JESTR offers a novel implicit annotation paradigm that avoids the explicit generation of spectra, fingerprints, or molecular structures. As molecules and spectra are views of the same object, embedding these views in a joint embedding space using contrastive learning provides a performance advantage. On NPLIB1, JESTR outperforms ESP by 88.9% and MIST by 41.1% on ranks@[1–5]. On NIST2020, JESTR outperforms ESP by 68.5% and MIST by 25.8% on average on rank@[1–5]. On MoNA, JESTR outperforms ESP by 57.5% and MIST by 4.0% on average on rank@[1–5]. Analysis of JESTR’s performance on the three datasets reveals that dataset diversity, quality, and spectra-to-molecule ratios impact performance. Further, our results showed enhance value in utilizing candidate molecules during training for regularization, improving performance an average of 11.4% for all three datasets. The overall JESTR results are promising and vouch for the potential of implicit annotation approaches. We expect to attain further improvements by utilizing additional knowledge in the form of subformulae annotation on spectral peaks and by utilizing enhanced molecular and spectral encoders.

## Supplementary Material

Supplement 1

## Figures and Tables

**Table 1.** Spectra, molecule, and candidate statistics for the three datasets.

	Total		Train				Test			

Dataset	Spectra	Molecules	Spectra	Molecules	Max Cands	Avg Cands	Spectra	Molecules	Max Cands	Avg Cands

NPLIB1	8,030	7,131	7,211	6,417	44,374	2,220	819	714	25,929	2,274
NIST2020	291,515	22,001	262,408	19,800	42,542	1,390	29,107	2,201	42,376	1,322
MoNA	35,752	6,767	32,216	6,090	42,542	2,322	677	3,536	32,364	2,494

**Table 2.** Ranking results for JESTR for the NPLIB1, NIST2020, and MoNA datasets. A. Ranking performance of all three tools B. Ablation study for the impact of removing regularization.

	NPLIB1			NIST2020			MoNA		
A. Comparison with other tools									
Model	Rank@1	Rank@5	Rank@20	Rank@1	Rank@5	Rank@20	Rank@1	Rank@5	Rank@20
ESP	23.7	44.7	60.2	20.5	30.1	48.8	19.4	38.4	53.0
MIST	27.9	62.4	82.1	21.0	57.4	78.8	**32.3**	52.9	75.3
JESTR (w/ regularization)	**45.8**	**81.5**	**95.8**	**38.6**	**59.6**	**81.0**	26.6	**64.2**	**91.2**
B. Ablation study									
JESTR (w/o regularization)	41.1	81.1	96.1	36.4	56.5	77.9	19.4	52.8	83.9
